# Supplementary material for: Natural plant disease suppressiveness in soils extends to insect pest control
Source: Microbiome. 2024 Jul 16;12:127. doi: 10.1186/s40168-024-01841-w (PMC11251354; doi:10.1186/s40168-024-01841-w)
Supplement: Supplementary file 2 — Supplementary file 1: Supplementary Fig S1. Differences in plant growth parameters between conditions. Supplementary Fig. S2. Changes in levels of defense-related phytohormones in wheat roots and leaves exposed to Oulema melanopus larvae. Supplementary Fig. S3. Changes in benzoxazinoid (BX) concentration in wheat roots and leaves exposed to Oulema melanopus larvae. Supplementary Fig. S4. Concentration of the cyanogenic compounds linamarin and lotaustralin in leaves of plants exposed to Oulema melanopus larvae. Supplementary Fig. S5. Microbiome composition and diversity of soils, wheat rhizospheres/phyllospheres and Oulema melanopus insects. Supplementary Fig. S6. Relative abundance of key bacterial taxa throughout samples. Supplementary Fig. S7. Network modularity and number of edges in the rhizosphere of wheat plants exposed or not to Oulema melanopus larvae. [file 40168_2024_1841_MOESM1_ESM.pdf]

Natural plant disease suppressiveness in soils extends to insect pest control

- Supplementary material -

Nadine Harmsen[1,2,†], Pilar Vesga[1,3,†], Gaétan Glauser[4], Françoise Klötzli[5], Clara M. Heiman[1], Aline Altenried[1], Jordan Vacheron[1], Daniel Muller[6], Yvan Moënne-Loccoz[6], Thomas Steinger[5], Christoph Keel[1]#, Daniel Garrido-Sanz[1]#

[1]Department of Fundamental Microbiology, University of Lausanne, Lausanne, Switzerland
[2]Current address: Institute of Earth Sciences, University of Lausanne, Lausanne, Switzerland
[3]Current address: Centro de Biotecnología y Genómica de Plantas, Universidad Politécnica de Madrid–Instituto Nacional de Investigación y Tecnología Agraria y Alimentaria, Madrid, Spain
[4]Neuchâtel Platform of Analytical Chemistry, University of Neuchâtel, Neuchâtel, Switzerland
[5]Agroscope, Research Group in Entomology, Nyon, Switzerland
[6]Université Claude Bernard Lyon 1, CNRS, INRAE, VetAgro Sup, UMR5557 Ecologie Microbienne, Villeurbanne, France
[†]These authors have contributed equally: Nadine Harmsen, Pilar Vesga

#Address correspondence to Christoph Keel (christoph.keel@unil.ch) and Daniel Garrido-Sanz (daniel.garridosanz@unil.ch)

The following supporting information is available for this article:

Supplementary Fig. S1. Differences in plant growth parameters between conditions.

Supplementary Fig. S2. Changes in levels of defense-related phytohormones in wheat roots and leaves exposed to *Oulema melanopus* larvae.

Supplementary Fig. S3. Changes in benzoxazinoid (BX) concentration in wheat roots and leaves exposed to *Oulema melanopus* larvae.

Supplementary Fig. S4. Concentration of the cyanogenic compounds linamarin and lotaustralin in leaves of plants exposed to *Oulema melanopus* larvae.

Supplementary Fig. S5. Microbiome composition and diversity of soils, wheat rhizospheres/phyllospheres and *Oulema melanopus* insects.

Supplementary Fig. S6. Relative abundance of key bacterial taxa throughout samples.

Supplementary Fig. S7. Network modularity and number of edges in the rhizosphere of wheat plants exposed or not to *Oulema melanopus* larvae.

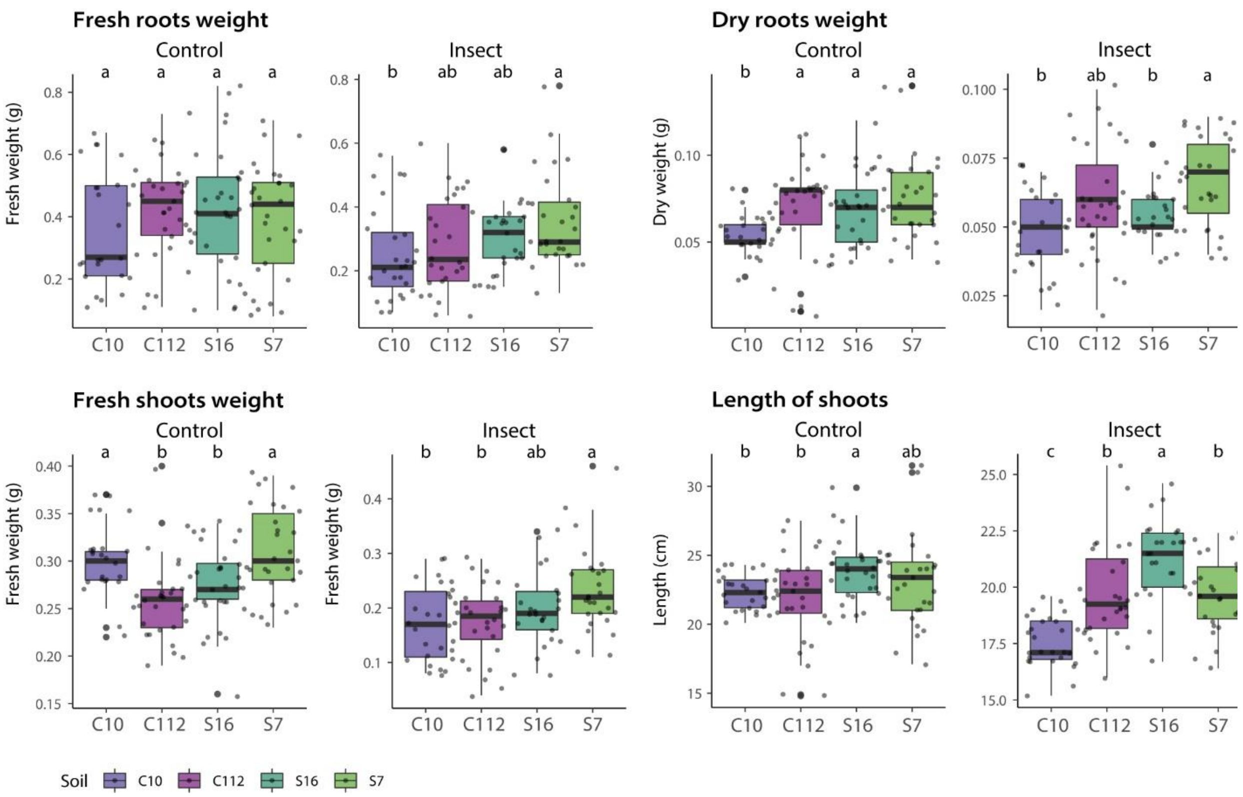

Supplementary Fig. S1. **Differences in plant growth parameters between conditions**. Boxplots show fresh/dry root and shoot weights and the length of fresh shoots for plants exposed or not to feeding by larvae of *Oulema melanopus*. The points represent individual replicates (n ≥ 25). Statistical differences were assessed using the Kruskal-Wallis test and LSD post hoc analysis. The *P* values were corrected by FDR. Different letters indicate significant differences between groups (*P* ≤ 0.05).

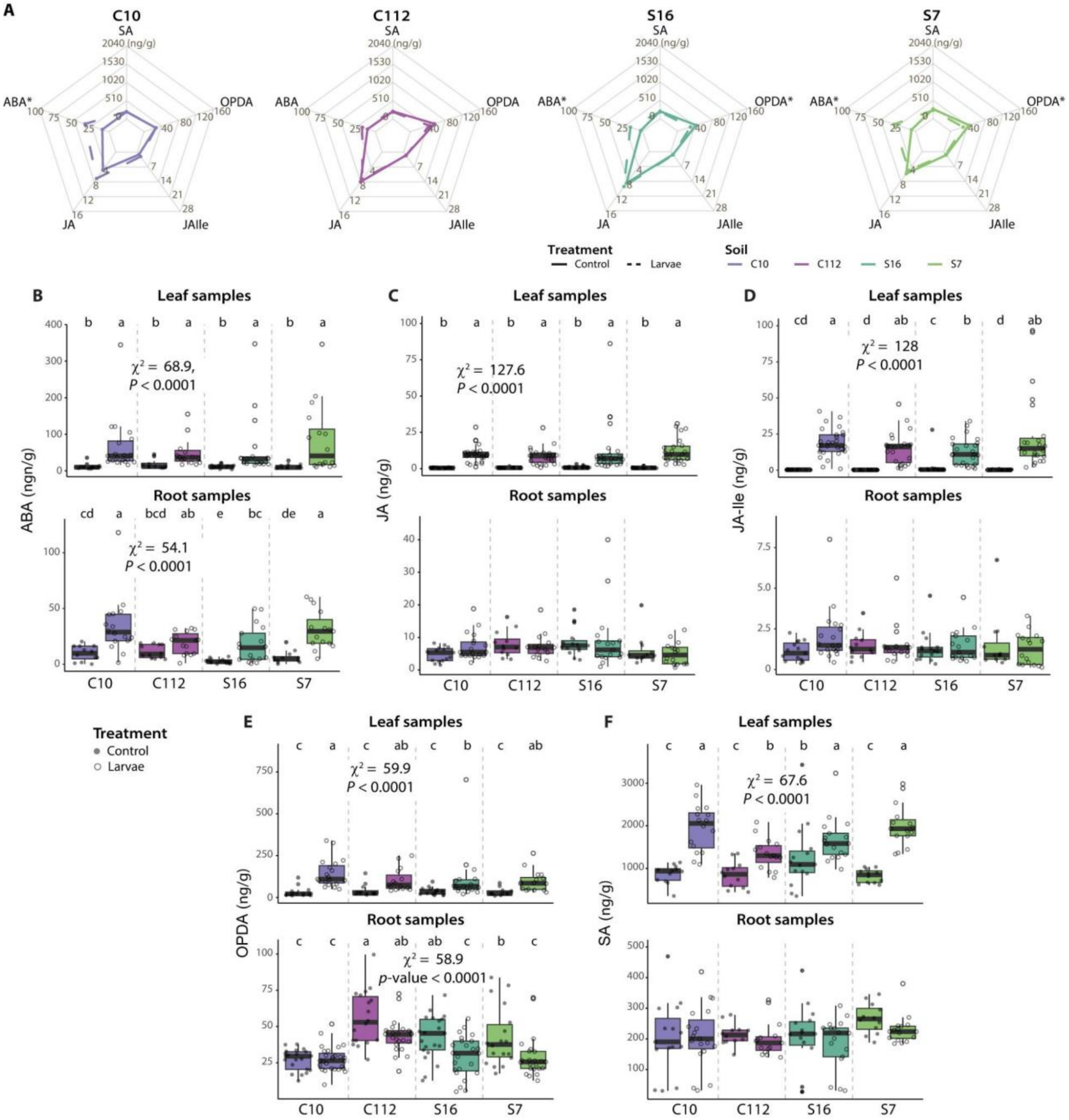

Supplementary Fig. S2. **Changes in levels of defense-related phytohormones in wheat roots and leaves exposed to *Oulema melanopus* larvae**. **A** Spider plots show insect and control group concentrations of abscisic acid (ABA), jasmonic acid (JA), jasmonyl-isoleucine (JA-Ile), 12-oxo-phytodienoic acid (OPDA), and salicylic acid (SA) in root samples in the soils C10, C112, S16, and S7. Asterisks denote phytohormones whose concentrations differed significantly between insect and control groups (Kruskal-Wallis, $P \leq 0.05$). **B-F** Boxplots representing the concentration of (**B**) ABA, (**C**) JA, (**D**) JA-Ile, (**E**) OPDA, and (**F**) SA in plants that had been exposed or not to the insect in the four soils ($n \geq 12$, at least two experimental runs were performed). The points represent individual replicates. Statistical differences were assessed using the Kruskal-Wallis test and LSD post hoc analysis. The *P* values were corrected by FDR. Different letters indicate significant differences between groups ($P \leq 0.05$).

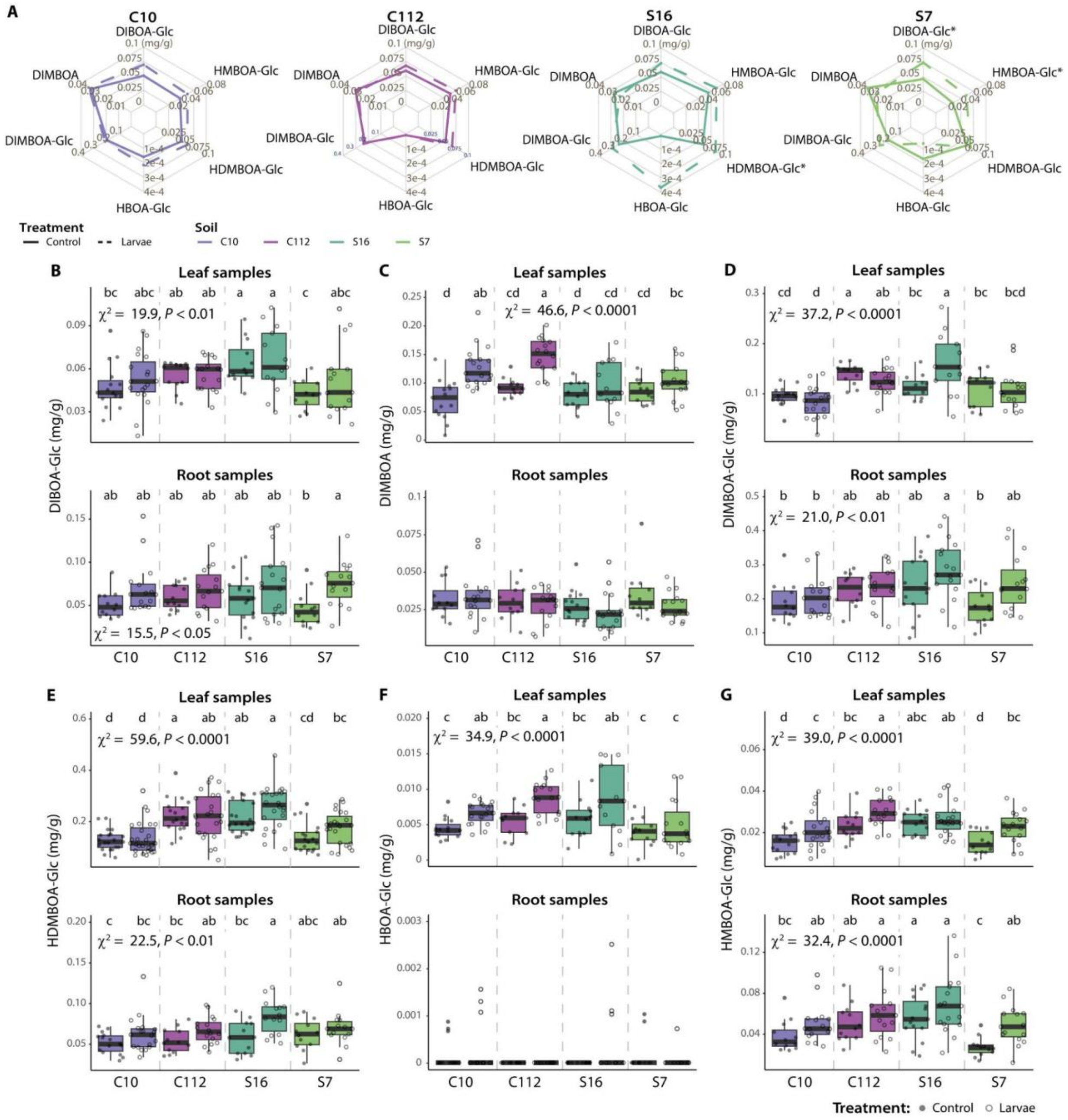

Supplementary Fig. S3. **Changes in benzoxazinoid (BX) concentration in wheat roots and leaves exposed to *Oulema melanopus* larvae**. **A** Spider plots show *O. melanopus* larvae and control group concentrations of DIBOA-Glc, DIMBOA, DIMBOA-Glc, HDMBOA-Glc, HBOA-Glc, and HMBOA-Glc in root samples in the soils C10, C112, S16, and S7. Asterisks denote BXs whose concentrations differed significantly between insect and control groups (Kruskal-Wallis, $P \leq 0.05$). **B-G** Boxplots representing the concentrations of (**B**) DIBOA-Glc, (**C**) DIMBOA, (**D**) DIMBOA-Glc, (**E**) HDMBOA-Glc, (**F**) HBOA-Glc and (**G**) HMBOA-Glc in root or leaf samples exposed or not to the larvae in the four soils ($n \geq 12$, two experimental runs for leaf samples, one for root samples). The points represent individual replicates. Statistical differences were assessed using the Kruskal-Wallis test and LSD post hoc analysis. The P values were corrected by FDR. Different letters indicate significant differences between groups ($P \leq 0.05$).

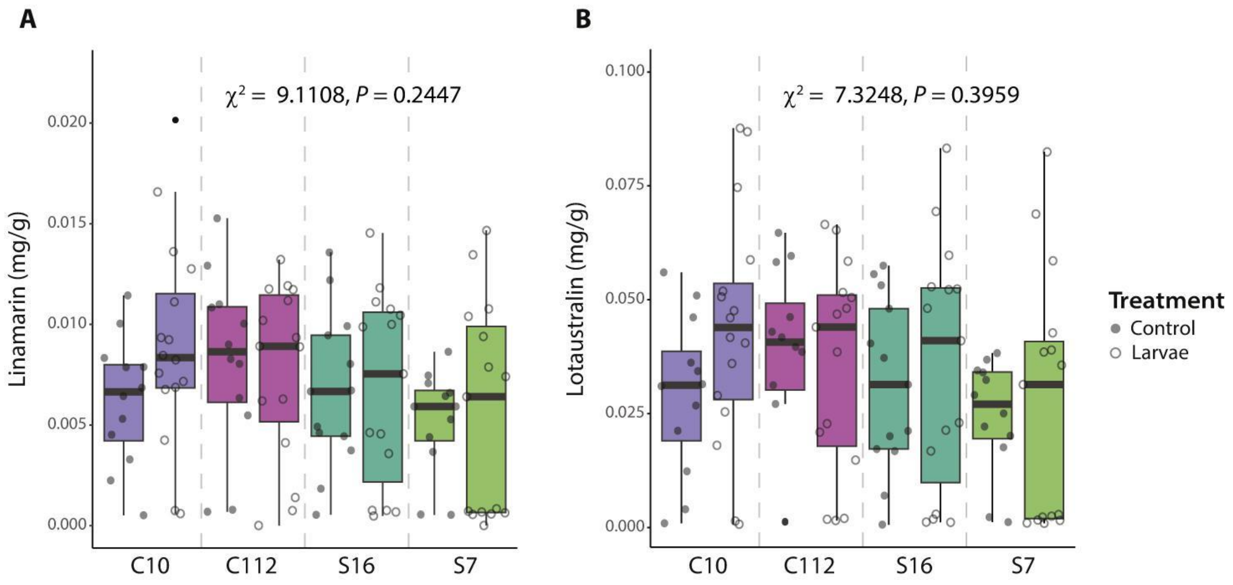

Supplementary Fig. S4. **Concentration of the cyanogenic compounds linamarin and lotaustralin in leaves of plants exposed to *Oulema melanopus* larvae**. **AB** Differences in the concentrations of (**A**) linamarin and (**B**) lotaustralin between plants growing with *O. melanopus* larvae and control plants ($n \geq 12$, one experimental run). Box plots showing the concentrations in mg per gram of tissue. The points represent individual replicates. There were no statistical differences ($P \geq 0.05$) between samples based on the Kruskal-Wallis test.

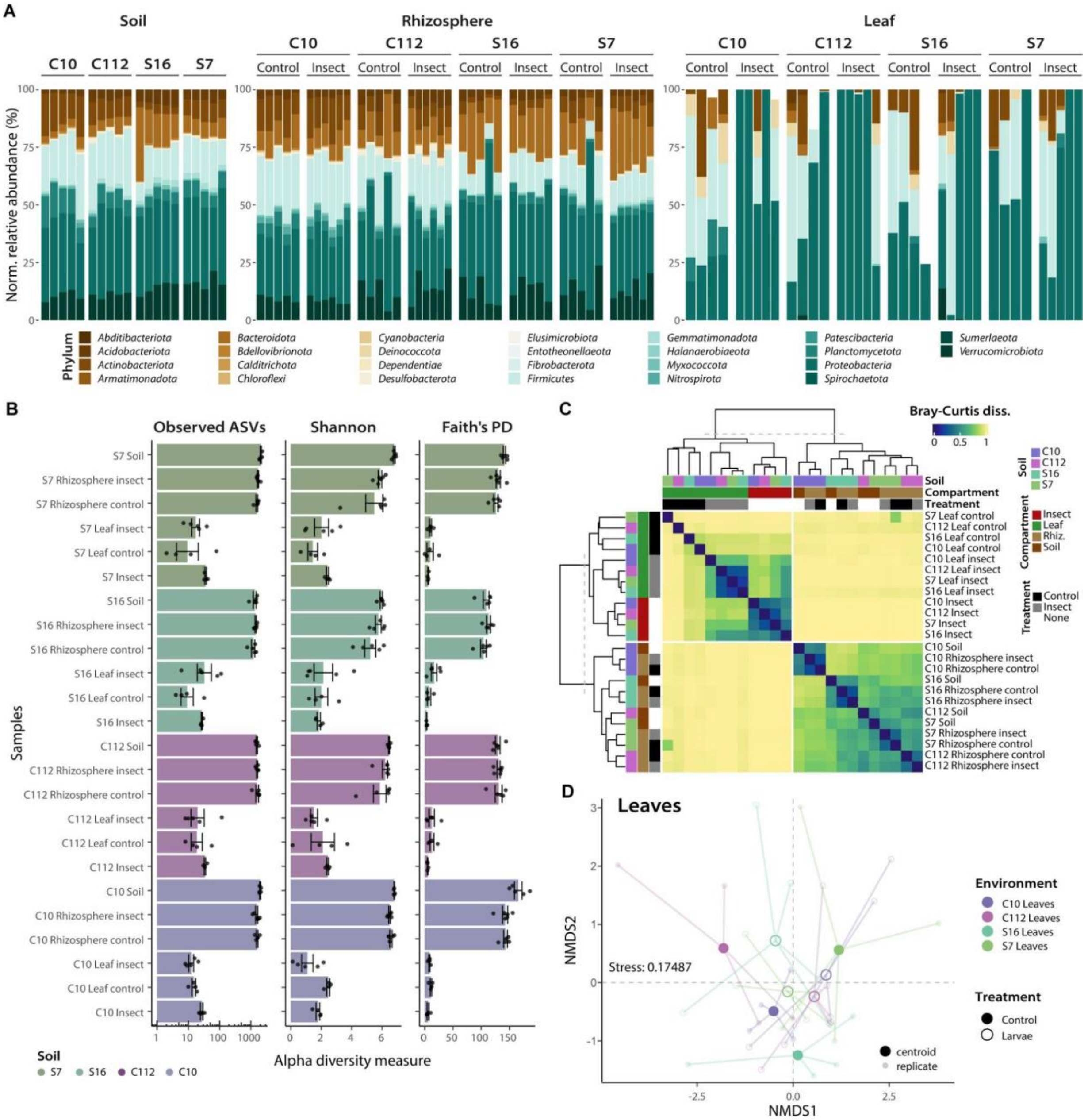

Supplementary Fig. S5. **Microbiome composition and diversity of soils, wheat rhizospheres/phyllospheres and *Oulema melanopus* insects**. **A** Cumulative sum scaling (CSS)-normalized relative abundance of ASVs in samples at the phylum level. **B** Alpha diversity measurements (observed ASVs, Shannon diversity and Faith's phylogenetic diversity (PD)) across samples. Bars represent mean values ± s.e. ($n \geq 4$). Black dots represent individual replicates. **C** Complete-linkage clustering of samples based on Bray-Curtis dissimilarities and heatmap. Replicates were merged using the mean ASVs abundance. **D** Non-metric multidimensional scaling (NMDS) ordination analysis of leaf samples based on Bray-Curtis dissimilarities.

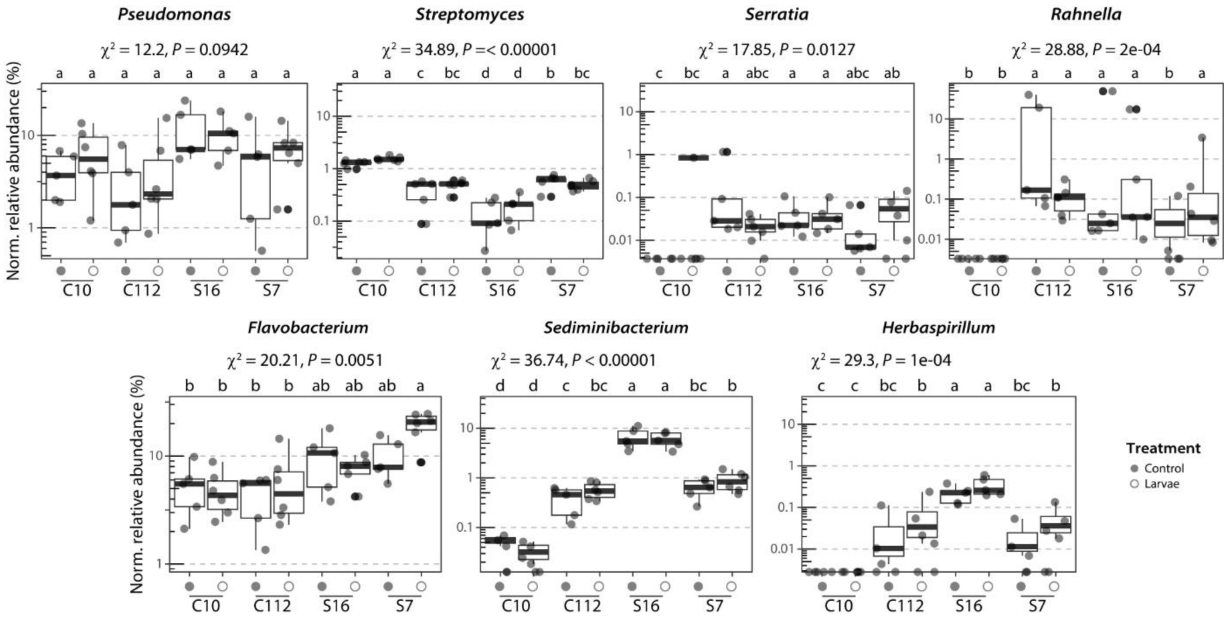

Supplementary Fig. S6. **Relative abundance of key bacterial taxa throughout samples**. CSS-normalized (Norm.) relative abundance of genera showing differential abundance changes in the rhizosphere of wheat plants growing in the different soils exposed or not to *Oulema melanopus* larvae. Dots represent individual replicates ($n \geq 4$). Dots collapsed at the bottom of the plots signify absence of the taxa (i.e., relative abundance = 0). Significant differences were assessed using the Kruskal-Wallis rank sum test and post hoc analysis by means of the Fisher's least significant difference. The P values were corrected by FDR. Different letters indicate significant differences between groups ($P \leq 0.05$)

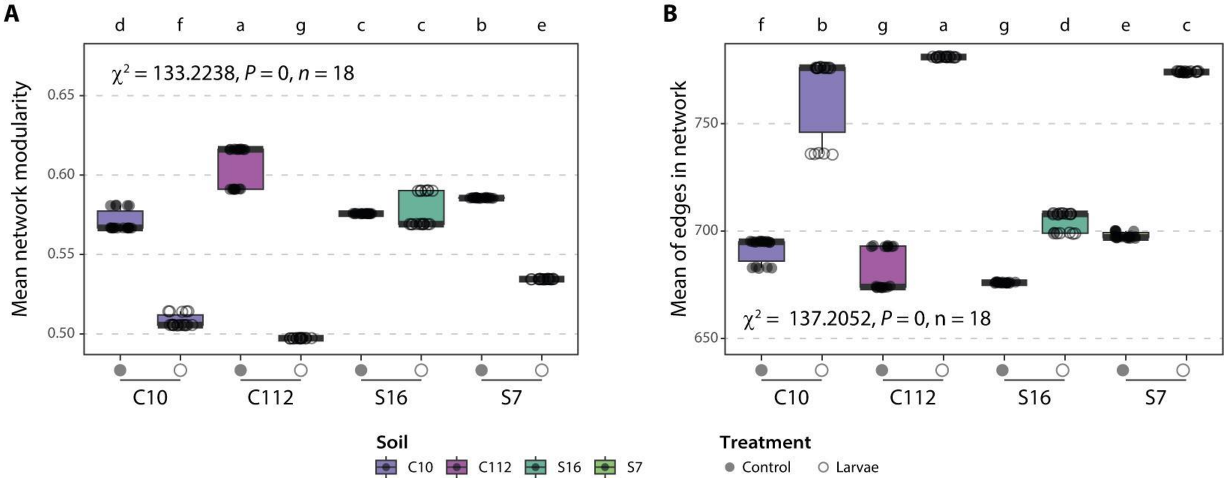

Supplementary Fig. S7. **Network modularity and number of edges in the rhizosphere of wheat plants exposed or not to *Oulema melanopus* larvae**. **AB** Mean network modularity (**A**) or mean number of edges (**B**) in association networks based on sparse inverse covariance estimation among the top 250 ASVs from rhizosphere microbiome samples in control plants or those exposed to *O. melanopus* larvae herbivory across the four soils studied. Statistical differences were assessed using the Kruskal-Wallis test and post hoc analysis using Fisher's least significant difference. The *P* values were corrected by FDR. Different letters indicate significant differences between groups ($P \leq 0.05$).
